# New frontiers for unmanned aerial vehicles in planetary health research

**DOI:** 10.1186/s44263-026-00250-5

**Published:** 2026-03-13

**Authors:** Juliet T. Bramante, Morgan S. Tarpenning, Katherine E. Woo, Andrew J. Chamberlin, Kavita D. Coombe, Joelle I. Rosser

**Affiliations:** 1 School of Medicine, University of Washington, Seattle, WA USA; 2 Stanford University, Stanford, CA USA; 3 Department of Earth System Sciences and Department of Oceans, Hopkins Marine Institute, Stanford University, Stanford, CA USA; 4 Division of Infectious Diseases & Geographic Medicine, School of Medicine, Stanford University, Stanford, CA 94305 USA

**Keywords:** Unmanned aerial vehicle, Drone, Climate change, Planetary health, Healthcare systems, Remote sensing

## Abstract

Unmanned aerial vehicles (UAVs) are a revolutionary new surveillance and transport technology with important implications for healthcare systems, particularly in the era of climate change. Rapid shifts in environmental systems are reshaping global climates. These changes have led to increasingly common extreme weather events that threaten population health. Mitigating the impacts of climate change on human health depends on our ability to predict, detect, and rapidly respond to changing ecosystem dynamics. The use of UAVs to tackle these new environmental health challenges is gaining momentum across multiple disciplines. This review identified four main areas where UAVs are being used or piloted to address climate change and health-related concerns: (1) Disease vector management, (2) environmental risk factors management, (3) environmental resource management, and (4) medical deliveries. Over the coming decades, UAVs are likely to play an increasing role in our efforts to keep pace with monitoring and mitigating the accelerating impacts of climate change on human health.

## Background

The effects of climate change are increasingly evident in shifting weather patterns, extreme weather events, and environmental disasters [1]. These climate changes affect the stability of natural systems that support human populations, contributing to complex and interacting health risks [2]. The field of planetary health aims to understand and act upon these relationships between the planet’s climate systems and human activities in order to protect human health. As defined by the Rockefeller Foundation–Lancet Commission, “planetary health is the health of human civilization and the state of the natural systems on which it depends [3].” As environmental stressors escalate, there is a growing need for flexible tools capable of supporting planetary health research and intervention. Unmanned aerial vehicles (UAVs) are proving to be useful tools for a wide variety of health-related applications in the face of these hazardous and often unpredictable environmental changes.

UAVs offer unique advantages as both a surveillance and a transport technology compared to traditional aircraft, ground-based approaches, and satellite imaging. UAVs are light, maneuverable, and low cost per unit compared to traditional aircraft. They can also be customized with specific sensor or delivery capabilities [4]. In surveillance, UAVs offer the ability to efficiently cover large, inaccessible, or otherwise infrequently surveyed areas of interest that are critical to planetary health monitoring and can do so more frequently and with greater detail compared to traditional survey methods [5]. Ground-based approaches are often time-, labor-, and cost-intensive and in some cases dangerous [6–8]. Ground observations can also miss important objects or spatial relationships that are not visible from the ground path [9, 10]. UAV-based imaging can provide pixel resolution of less than 0.5 cm/pixel depending on the sampling distance or height of the UAV flight [11]. This image quality surpasses even very high-resolution satellite data, which ranges from 30 cm/pixel to more commonly several meters/pixel [12]. Satellite imagery also can be affected by poor visibility and cloud cover, whereas UAV imaging can be deployed on a flexible schedule and from strategic launch points to avoid high winds and other adverse weather.

As a transport and intervention delivery technology, UAVs can sometimes take more efficient routes to targets, which is particularly advantageous for applications in remote areas or in disaster-affected regions where ground infrastructure is damaged [7]. In these situations, replacing human couriers with UAVs can reduce dangerous human exposures and maintain supply chains while supporting planetary health interventions. Additionally, many UAVs are electric and reduce reliance on fossil fuels. Although less commonly used due to onboard computation constraints, real-time surveillance enables UAVs to directly intervene or issue detection alerts during flights [13, 14].

The effective use of UAVs is in many cases contingent on pilot expertise, high-quality ground data for validation, and permissive weather conditions for flights. UAVs are versatile tools for imaging and transportation, which can be leveraged to provide a novel perspective in a wide range of contexts. However, each unique application presents specific challenges and opportunities for further innovation. The aerial perspective of UAV imaging can provide valuable visuals that are not within line of sight from the ground, but UAVs are not a complete substitution for ground surveys. Target objects may be indistinguishable or obscured on UAV images, and thus, UAV observations should be validated against ground surveys [6, 15]. Flying UAVs is also more time- and resource-intensive than other forms of remote sensing such as satellite imaging, which can repeat measurements of a larger surface area more frequently [16]. Delivering supplies with UAVs without the accompaniment of trained personnel is not appropriate for all situations and supply types [17]. Moreover, in denser urban areas, UAV transport may pose risks of collision, interference with other flight systems, and technology failures leading to unsuccessful or inaccurate supply drop-off [18]. These challenges underscore the need to carefully consider contexts in which UAVs offer a comparative advantage over traditional methods or where UAVs can be used to complement or augment current strategies.

**Fig. 1:**
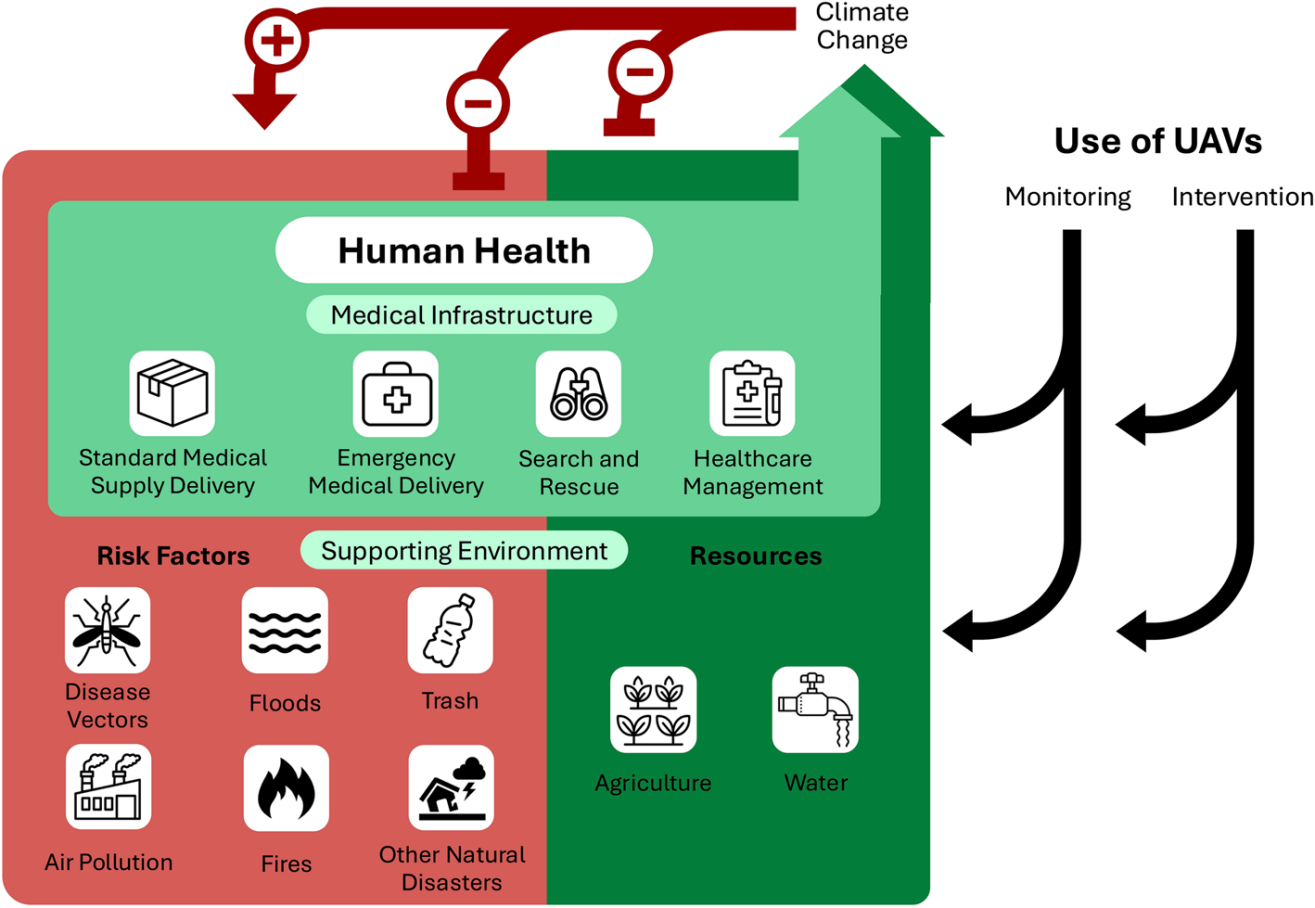
Framework for understanding human health in the context of climate change and the potential role of UAVs. Human health is supported by structured systems that provide standard and emergency healthcare and maintain healthcare supply chains. These infrastructures depend on stability in surrounding ecosystems, which provide key resources including agriculture and water but also pose risks to human health that include vectors for disease, floods, trash, fires, air pollution, and other natural disasters. Climate change is damaging supportive systems and exacerbating risk factors. UAVs can be used for both surveillance and intervention to support both healthcare infrastructure and management of environmental risks and resources

Existing review papers focus on the environmental applications of UAVs, as well as their emerging use in human health contexts, but interdisciplinary discussion of UAVs in planetary health remains limited. Prior literature examines pilot studies or reviews a single planetary health domain, such as fire management, pollution monitoring, or ethical implications. The goal of this review is to provide a broad overview of current applications, limitations, and future directions for the use of UAVs within a planetary health framework. Here, we evaluate UAV technology as a tool for monitoring and mitigating ecosystem disruption, human injury, and disease in the context of climate change. We focus on four main applications for UAVs in addressing climate change and health-related concerns: (1) Disease vector management, (2) environmental risk factors management, (3) environmental resource management, and (4) medical deliveries (Fig. 1).

## Vector-borne disease (VBD) monitoring and management

### Surveillance of vectors and disease risk

VBDs are diseases transmitted to people by intermediary hosts such as mosquitoes, ticks, or snails and include diseases like malaria, dengue, schistosomiasis, Chagas disease, Zika, leishmaniasis, and onchocerciasis. Worldwide, there are over 300 million reported cases and 700,000 deaths annually due to VBDs [19]. Vectors typically flourish under specific environmental conditions, and mapping the presence of characteristic habitats can help to identify high-risk areas. Moreover, monitoring changing ecological habitats and networks in response to climate change, deforestation, and urbanization can help predict shifts in disease risk [19].

UAVs are a promising and rapidly evolving tool for mapping suitable habitats and high-risk areas for VBDs. While the majority of research has focused on dengue and malaria vectors, UAVs are increasingly being used to study other VBDs (Table 1). The accuracy and efficiency of UAV-based approaches compared to traditional surveys are variable. UAVs can identify more potential habitats [28], cover a greater area compared to traditional ground surveys [6], and visualize features like vector habitats on rooftops. However, buildings and vegetation can obstruct aerial visualization [4], and camera resolution may still be inadequate to identify very small features [29]. Ultrahigh-resolution UAV imaging, on the scale of mm/pixel, has been used in detailed agricultural monitoring and exhibits the potential for variable image resolution depending on contextual needs [30, 31]. The achievable accuracy and spatial scale of habitat classification are influenced by UAV and camera specifications, local flight height regulations, habitat complexity, protocol choices, and classification needs. As a result, UAV-based approaches must be tailored to the specific epidemiological question and vector ecology to ensure accurate classification and risk assessment.

**Table 1:** UAV-based approaches to vector-borne disease surveillance

Disease	Vector	Preferred habitat	Methods	Classification strategy	Results	Setting	Authors
Dengue, chikungunya, and Zika viruses	*Aedes aegypti*	Small-scale standing water bodies including man-made containers (tires, barrels, plastic drums, stagnant drains, plastic containers, puddles, trays)	Manual identification from UAV-based images	Discrete categories of containers	The UAV-based approach identified 1 container per 2.8 identified by ground surveillance but more frequently identified backyard and roof containers	Mexico	Valdez-Delgado et al. (2021) [9]
				Discrete categories of trash	Development of a trash identification scheme based on likelihood of producing an *Ae. aegypti* breeding site, comparison of UAV images to ground-based surveys	Kenya	Rosser et. al. (2024) [15]
			CNN for object detection	Discrete categories of containers: water tanks and swimming pools	Areas with low socioeconomic status (SES) had more exposed water tanks, and areas with high SES had more swimming pools	Brazil	Cunha, H. S. et al. (2021) [5]
			Logistic regression with UAV-derived characteristics	Multiple data streams: visible water containers, vegetation indices (GRVI)	More vegetation near the household was associated with more dengue (OR: 11.3, 95% 0.38, 38.0), as was proximity to a football field (OR: 13.9, 95% 4.0, 48.4)	Ecuador	Lee, G. O. et al. (2021) [10]
	*Aedes albopictus* (Skuse)	Small (centimeter scale) water-holding containers	CNN for object identification (SSD300)	Discrete objects of interest	Up to 67% precision and 40% recall for neural network identification of containers on UAV images, accurate prediction of whole properties as positive or negative for larva with 80% frequency	USA	Case, E. et al. (2020) [6]
			CNN for image classification (YOLO v7)	Discrete categories of common containers	The model achieved an F1 score and mean average precision (mAP) both above 0.99, indicating high performance. Community-wide, 97% of potential breeding sites were located correctly. The model correctly predicted the houses with highest container density	China	Yu, K. et al. (2024) [20]
Malaria	*Nyssorhynchus darlingi*	Water bodies with sunlight and vegetation	Random forest classification with multispectral data and vegetation indices (NDVI)	All bodies of water, visual index for vegetation or water depth	Ability to identify water bodies where *Ny. darlingi* is most likely to breed with an overall accuracy of 86.73%–96.98%	Peru	Carrasco-Escobar, G. et al. (2019) [21]
	*Anopheles gambiae*	Small or transient sunlit small pools	Manual identification	All bodies of water	Proof-of-concept use of UAV images for larval source management planning. Found to be low cost and flexible (higher up-front equipment costs, cheaper with repeated surveys)	Tanzania	Hardy, A. et al. (2017) [22]
	*Anopheles arabiensis*						
	*Anopheles funestus*	Larger, permanent bodies of water, often with vegetation	Manual identification	Classification scheme based on breeding ecology	Technical workflow for integration of UAV surveys and entomological sampling. All sampled water bodies were identifiable in UAV imagery	Côte d’Ivoire	Byrne, I. et al. (2021) [23]
	Combined vector approach (*An. gambiae*, *An. arabiensis*, *An. funestus*)	Varied	Geographical object-based image analysis (GeoOBIA) and random forest classification, ±near-infrared (NIR) data	All bodies of water and aquatic vegetation	High accuracy in identifying larval habitat characteristics (median accuracy of 98%), no notable improvement with addition of NIR data	Malawi	Stanton, M. et al. (2021) [24]
			CNN classification (U-Net and attention U-Net)	Ground cover classifications including vegetated and non-vegetated bodies of water	Dice coefficients of 0.68 for vegetated and 0.75 for non-vegetated water bodies, 0.88 for tillage and crops, 0.87 for buildings, and 0.71 for roads indicate varying levels of agreement with reference data across categories	Burkina Faso and Côte d’Ivoire	Trujillano, F. et al. (2023) [25]
Schistosomiasis	Freshwater snails	Aquatic vegetation	CNN classification (U-Net), comparison to random forest	Ground cover classifications (floating vegetation, emergent vegetation, water, and land)	The CNN performed better than random forest at identifying all types of land cover and vegetation alone. CNN classification of the validation set of images resulted in an accuracy of 82.7% or 83.1% (added textural analysis) for all four types of ground cover and 84% for vegetation which is the most critical target	Senegal	Liu, Z. et al. (2022) [26]
Chagas disease	*Triatoma dimidiata*	Mixed domiciliary and ecological factors	Automatic classification with ArcGIS Pro 2.5 algorithm	Land use classifications (houses, monocultures and pastures, woodland and shrubland, and bare soil)	Domiciliary factors (wall and floor composition, animal presence, clutter) collected by survey were associated with infection risk, and land use was not — this was attributed to high levels of deforestation at all study sites	Guatemala	Penados et al. (2020) [27]

UAVs are being used to identify habitats of various VBDs, with accuracy aff ected by local context and methodological choices. A variety of classification methods can be employed depending on the target of interest

*Abbreviations*: *UAV* unmanned aerial vehicle, *CNN* convolutional neural network (a deep learning algorithm), *GRVI* Green-redvegetation index, *NDVI* Normalized difference vegetation index

### Deployment of interventions

UAVs are increasingly being deployed to improve larval source management strategies. UAVs have already been used to release sterile male mosquitoes, *Wolbachia*-infected mosquitoes, and pesticides — three methods currently used to control mosquitoes that transmit dengue and malaria.

Several advantages of UAV versus ground deployment of mosquitoes and pesticides have been identified. Deployment of sterile mosquitoes and *Wolbachia*-infected mosquitoes are currently being studied as promising methods to reduce populations of disease-carrying mosquitoes [32–34]. UAV deployment has been found to yield quicker and more homogenous deployment, reduce need for technicians, and reduce discomfort for communities compared to ground deployment of mosquitoes [35]. UAVs have also been used to aerially deploy pesticides against *Anopheles* and *Aedes *mosquitoes [28], the biologic agent *Bacillus thuringiensis* against *Aedes vigilax *(Skuse) mosquitoes [36], and larvicidal film against *Anopheles arabiensis* [37]. UAVs have been shown to reduce the routine operational costs of larval source management, primarily through decreased personnel requirements. In Zanzibar, the implementation of UAV-based larviciding required 1102 labor hours, compared with 1603 labor hours for conventional ground-based methods. Despite reductions in labor, the cost per person protected and cost per area covered were comparable across approaches, largely due to the initial cost of community engagement programming to ensure public UAV acceptance [28]. The rising availability and decreasing costs of UAVs may lead to more widespread adoption of this approach in the future [28].

Further development of protocols and advances in UAV technology will provide a wider range of use cases in VBD monitoring and management. Standardizing vector habitat quantification and risk assessment using UAV imaging will enable comparison between time points and geographical regions. This standardization will likely include exploring optimal image resolution, flight height, and multispectral wavelengths for the vector or disease of interest. Additionally, it may be beneficial to develop guidelines that direct strategic ground surveys to augment the UAV images, for example, targeting areas where dense tree or roof cover may be obstructing the aerial identification of risky habitat. Continued data validation through ground surveys across diverse contexts may enable future UAV-based risk assessment in remote areas where traditional ground approaches and validation are not possible.

There are also opportunities for technological development to improve the deployment of interventions in infectious disease applications. One potential issue found with UAV release of sterile mosquitoes is that the number of successfully released mosquitoes may be reduced due to damage in transit. The mosquitoes that are released, however, appear to be just as competitive [38]. Advancing temperature control during transport and other vector-protecting measures could increase the successful yield using UAV deployment. Unique and changing local regulations regarding UAVs also continue to present an obstacle to widespread use. There is a persistent need, therefore, to gain community understanding and acceptance of UAV technology in VBD management contexts.

The emerging use of UAVs in planetary health presents an exciting frontier, with the potential to shed new light on infectious disease dynamics as environmental changes bring unforeseeable shifts. Moreover, UAVs serve as a novel tool for VBD mitigation, both through direct deployment of interventions as well as surveillance to target and measure the impact of vector habitat abatement measures.

## Management of environmental risk factors

As climate change continues, natural disasters like floods and wildfires are increasing in frequency and severity, resulting in both direct and indirect impacts on planetary health. Furthermore, worsening air pollution and garbage accumulation compound many of the health impacts of climate change. UAVs offer novel approaches to monitoring and mitigating these environmental risk factors.

### Floods

Flooding poses an increasing risk in many parts of the world as climate change drives heavier rainfall, more severe storms, and rising sea levels [39]. Beyond the immediate risks of injury and death, floods create cascading health hazards including increased disease transmission and disrupted access to healthcare and essential resources. Flooding can overwhelm sewage systems and household sanitation infrastructure, and affected populations may be forced to live in temporary settlements with crowding, poor sanitation, and inadequate access to drinking water. These conditions commonly fuel outbreaks of diarrheal diseases such as typhoid and cholera [40]. Transmission of nondiarrheal infectious diseases can also be exacerbated by floods. Pools of stagnant water left behind after flooding serve as breeding grounds for mosquitoes, which facilitate dengue transmission [40]. Floodwaters can also increase exposure to leptospirosis, transmitted through skin contact with flood water contaminated by the urine of infected rodents and dogs [40].

UAVs can be used both for early warning—to predict and detect floods—and for emergency response. High-resolution UAV images improve the accuracy of hydrological models of water movement for flood forecasting and enable automatic detection of flooded areas [41]. During and after floods, UAVs can support community evacuation plans, damage assessment, reconstruction, search and rescue, or other emergency responses [42]. UAVs offer notable advantages over satellite imaging, which has poor resolution at the household level and fixed revisit times that could miss crucial time periods. Although both UAVs and satellites face weather challenges during flooding periods, UAVs can be strategically deployed between storms and under clouds to collect data when satellite imagery is obscured. A project investigating flood damage at a levee of Poyang Lake, the largest freshwater lake in China which flooded in 2020, was able to detect flooded buildings and vegetation in UAV images with an accuracy of 88% and 85%, respectively [43].

### Trash

Solid waste has been associated with increased risk for many VBDs such as dengue, chikungunya, Zika, leishmaniasis, Chagas disease, and malaria, as well as zoonotic diseases including leptospirosis, scrub typhus, plague, toxoplasmosis, and rabies [44]. Dengue, chikungunya, and malaria are all associated with specifically the presence of rainwater-retaining litter around households, which creates breeding habitat for mosquito vectors [45]. Accumulated trash and proximity to open sewers or garbage disposal areas also increase the density of rat populations and subsequent risk of leptospirosis [40]. Flooding, as discussed above, can exacerbate these risks by disrupting trash collection, filling trash with water that breeds mosquitoes, and moving trash (and urine from leptospirosis-infected rodents) into closer contact with humans. For these reasons, garbage accumulation and compounding climate change characteristics pose a significant threat to human health.

There is early interest in using UAVs for real-time, automatic trash detection to improve the efficiency of waste management systems, although most activity in this area appears to still be hypothetical or proof of concept [13, 14]. UAV-based trash detection systems have been piloted in both urban [15] and agricultural settings [46].

### Air pollution

Air pollution is a major contributor to morbidity and mortality. While most frequently associated with cardiopulmonary conditions, it is increasingly recognized as a determining factor in a variety of health outcomes and as one of the greatest environmental risks to health [47]. Pollutants, which are commonly co-emitted with greenhouse gases, both currently contribute to climate change and may increase indirectly as a result of increasing global temperatures [47].

UAVs present a promising alternative or complement to traditional fixed ground-based sensors and satellites for detecting pollutants. Small, low-cost sensors designed to detect a variety of pollutants have been successfully mounted on UAVs and used to monitor natural pollutant levels from volcanoes and melting permafrost, urban air quality, fossil fuel mining and management, and byproducts from wastewater and agriculture [48]. Ground-based sensors can provide highly accurate measurements but are often expensive and immobile, allowing for close monitoring of a constrained area or of a larger region with scattered coverage. Satellite or manned-aircraft-based approaches lack spatial resolution but provide consistent, broad coverage that is well suited to track global patterns and seasonal changes. However, these approaches provide limited information on lower altitudes [49] and can face inaccurate readings over reflective land cover and during environmental disaster events [50]. UAVs, by contrast, can collect frequent fine-scale three-dimensional data on pollutant distributions across the lower atmospheric column and can also be directed to dangerous pollutant sources. However, a fully operational, large-scale air quality monitoring system using UAVs has yet to be implemented. Developing such systems will require the application of rapidly evolving technologies including network support for multiple UAVs, communication between UAVs, accurate, lightweight, and long-lasting sensor technology, and real-time changes in flight paths based on sensor readings [49].

### Fires

Fires pose immediate danger to the health of first responders and displaced persons while also worsening air pollution and disrupting ecosystems and infrastructure [51]. Rising global temperatures drive more frequent and severe droughts and heat waves, which in turn worsen the severity and frequency of wildfires [39, 52].

UAV-based fire surveillance has notable advantages over satellites, which have limitations in spatial resolution and ground visibility, and over remote wireless networks, which must be deployed ahead of time and are destroyed during the fire [53]. With their ability to perform surveillance or interventions in high-risk areas, UAVs are uniquely able to reduce risks to human life [8]. They are actively being used for fire detection and monitoring efforts in the USA, Canada, Australia, and Europe [8]. Multiple sensor and machine learning-based approaches are currently under investigation, including the use of thermal sensors and automatic detection of flames and smoke [8]. Preliminary research and development efforts are also exploring the use of UAVs to actively fight fires [54]. Some notable challenges in this area include coordination between UAVs, obstacle detection and avoidance, and flight path decision-making. Sensor and camera accuracy, battery power, GPS accuracy, and weather resilience are also important areas for improvement [8].

### Other natural disasters

UAVs have also been deployed in response to hurricanes, earthquakes, landslides, and rockfalls. In recent decades, climate change has increased the frequency and severity of natural disasters, with an estimated annual cost of $143 billion attributable to climate change, and 63% of this cost estimate is driven by loss of human life [55].

In these contexts, UAVs have been successfully applied to mapping and response planning, search and rescue, and delivery of supplies [7]. They can assess damage quickly and can be directed to otherwise inaccessible areas [7]. UAV images from World Food Program (WFP) have been used to plan disaster response efforts in multiple settings including cyclones in Mozambique in 2019 [56] and hurricanes in Dominica in 2017 [57], providing rapid assessments of safety and infrastructure. The WFP is also currently piloting UAV delivery of emergency supplies. The Japan Unmanned Aerial System Industrial Development Association partnered with the Japanese military to deliver supplies to displaced people who lost their homes during an earthquake in Japan in 2024. Streamlining UAV flights and coordinating UAVs with supporting ground networks are active areas of research [58].

Targeted UAV monitoring has demonstrated benefits across diverse environmental risks. However, a common obstacle to large-scale monitoring is the coordination of a network of UAVs to cover greater areas. This challenge is especially pronounced during and after natural disasters when communication, power, and Internet networks are unreliable and in high demand. UAV imaging may therefore be most effective for targeted regions or time periods of interest. Accordingly, integration with complementary sensing methods, such as satellite or stationary ground sensors, could enable the focused deployment of UAVs for more detailed data collection. Incorporating UAV technology into broader data collection workflows offers an opportunity to leverage the strengths of each method and warrants further exploration in environmental risk monitoring (Table 2).

**Table 2:** Comparison of unmanned aerial vehicles, satellites, and ground methods

	**Unmanned aerial vehicles**	**Satellites**	**Ground methods**
*Visualization and spatial accuracy*	Centimeter to millimeter resolution possible Trade-off between image resolution and coverage area Views of places difficult to reach by foot or frequently obscured by cloud cover RGB bands are most common, but specialized sensors (e.g., multispectral, LiDAR) are available for specific applications Three-dimensional views Aerial view may not identify all features	Kilometer to meter resolution publicly available Multispectral data frequently available Aerial view and coarse resolution may not identify all features Frequent cloud cover can obscure visualization for optical sensors Three-dimensional views possible but more limited	Close-up visualization from multiple angles allows for highly accurate feature identification Three-dimensional views Cannot visualize hard-to-reach places (e.g., on top of roofs or trees, away from roads or footpaths) Allows collection of additional contextual data (e.g., odors, sounds, community input)
*Temporal accuracy*	Can generally control timing of deployment (weather permitting) Requires manual deployment to get serial imaging Battery life limitations can affect survey duration	Regular intervals available, often going back many years and with anticipated future images Revisit times range from weekly for free platforms (e.g., Landsat and Sentinel) to daily for commercial options (e.g., Planet Labs)	Can generally control timing of ground surveys (conditions permitting) Requires manual deployment to do serial assessments
*Delivery of interventions*	Can distribute interventions (e.g., mosquitoes, pesticides) evenly and in hard-to-reach places May be faster or less expensive than traditional aircraft or ground methods for some areas or circumstances Risk of damage to items during transit	Not possible	Ensures correct delivery, possibly with accompanying trained personnel Ability to deliver heavier payloads and those with specific packaging and transportation needs Generally relies on intact ground infrastructure
*Logistics*	Efficient coverage of a relatively large area Can provide images or access during dangerous conditions Review of images can be time-intensive Requires trained licensed pilots to fly, as well as coordination with local aviation authorities and airports Flights can be disrupted by weather Community concerns about privacy and safety Some areas have restrictions on flight zones and flight height Requires advanced technical skills (e.g., mission planning, orthomosaic generation, and modeling) and powerful computing resources Up-front equipment and training costs, low operating costs	Widely available platforms for image acquisition and analysis Offers ready-made products (e.g., land cover maps), lower technical barriers, and free cloud-based processing (e.g., Google Earth Engine) Lower resolution imaging reduces privacy concerns No deployment costs, but subscription-based access can be expensive depending on platform	Restricted access to some areas Hazardous conditions may damage ground sensors or make data difficult to retrieve Human deployment to some areas and in some conditions can be unsafe Can be very time- or cost-intensive depending on objectives

Differences across methods in spatial and temporal accuracy, capacity for intervention delivery, and logistical considerations relevant to planetary health research. Abbreviation: *LiDAR* Light Detection and Ranging

The inherent exposure to extreme weather, debris, and infrastructure damage in such scenarios makes UAV substitution for dangerous human labor particularly beneficial. These same factors, however, complicate UAV object detection and weather resilience, highlighting them as key areas for improvement. Investing in real-time UAV analysis and automated response could improve intervention efficacy during disaster situations when operator communication is limited.

Overall, UAVs have demonstrated utility in monitoring environmental hazards across water, air, land, and other disaster domains. With continued development, UAVs have the potential to prevent property loss, protect human life, and support recovery efforts.

## Environmental resource management

Food insecurity due to drought, changing seasonal rainfall, and pest activity is a potential health threat due to climate change. Although the indirect health effects of droughts are difficult to measure, droughts are strongly correlated with malnutrition, injuries, cardiopulmonary conditions, and the spread of infectious diseases [59]. Changes in average temperatures are associated with increases in both moderate and severe food insecurity, and the effect appears to be intensifying with time [60]. Extreme weather events pose an additional risk for food security. Exposure to once-per-100-year extreme climate events may lead to a risk of hunger for an additional 11–33% or 20–36% of the global population by 2050 under low and high emission scenarios, respectively [61]. Policies aimed at reducing emissions, such as carbon taxes, may also contribute to food insecurity through their effects on the agricultural industry, a sector that together with land-use change accounts for roughly one-quarter of greenhouse gas emissions [62]. Optimizing food production is critical for climate change resiliency worldwide. UAVs can be used in this planetary health context to monitor the health of crops, water sources, and overall ecosystems.

### Agriculture

Potential applications for UAVs in agriculture include use of UAV images to monitor the health of crops, especially after fluctuations in water [63]. UAVs have a particular advantage for use in remote sensing because they provide systems-level visual data (e.g., of an entire crop) at a resolution in which the health of individual plants can be easily assessed [64]. Many indices have been proposed to analyze vegetation health and drought severity using either site-based or remote-sensing approaches. The suitability of these indices remains an active area of research [63]. In addition to monitoring crop health, UAVs are also being deployed for seeding crops and administering pesticides [65].

### Water management

UAVs can monitor water sources to support ecological health and agricultural, industrial, and domestic needs. Applications include assessing water quality metrics in small reservoirs [66] and pollution in urban rivers [67]. UAV-based monitoring is emerging as a cost-effective and efficient alternative to traditional survey methods [68]. The use of UAVs to monitor groundwater remains understudied, particularly in the Global South, yet presents an economically and ecologically promising field of study [68].

The application of UAVs for environmental resource monitoring has gained traction and brought about innovations in the quantity, quality, and affordability of data that can be extracted from UAV sensors. Scaling UAV monitoring operations remains a central challenge. As in environmental risk management, UAVs are well suited for surveillance of individual bodies of water or crop fields but may benefit from coordination with broader scale satellite monitoring to direct the high-resolution UAV imaging to specific regions or time points of interest. Local regulations specifying flight height, weight of UAV payloads, and other constraints are also difficult to navigate for consistent monitoring. Flight efficiency also intrinsically limits UAV weight and points to an ongoing need for lighter sensors capable of collecting diverse data types for agricultural and water assessment.

Moreover, collecting sufficient high-quality data to train models and inform UAV image analysis and classification has been highlighted as an obstacle in groundwater management. This challenge is especially acute in regions with fewer resources that will likely experience disproportionate climate change impacts and thus may benefit substantially from innovative monitoring techniques.

## Medical deliveries

UAVs are increasingly being integrated into both emergency medical systems and standard delivery of medical supplies. In this setting, UAVs have the advantage of bypassing ground infrastructure to maintain essential supply chains and reach targets in more rural or isolated areas. Pilot delivery projects cite reductions in carbon footprint as an advantage of UAVs compared to traditional delivery systems, depending in part on the energy requirements of the UAV, the delivery distance, and the available alternative transportation methods [69]. This flexibility and energy efficiency are particularly valuable in the context of climate change, where extreme weather events may make ground infrastructure less reliable.

### Standard medical supply delivery

Pilot or proof-of-concept studies have been performed for UAV delivery of chemotherapy, blood products [70], and vaccines including COVID-19 [71]. While pilot studies continue, there is increasing evidence of scaled commercial adoption, including sustained government contracts and revenue-backed growth. The company Zipline [72], which began text-based blood deliveries in Rwanda in 2016, expanded to deliver 20% of the country’s blood supply outside of the capital by 2019 and has since expanded delivery operations to Ghana, the USA, and Japan. The company Matternet has flown over 1800 flights in Switzerland and additionally has delivered medications in Haiti, the Dominican Republic, and New Guinea and has collaborated with UNICEF and Doctors Without Borders [73]. Using UAVs for vaccine delivery was estimated to reduce the delivery cost by up to $0.21 per dose administered, as compared to traditional methods [74].

### Emergency medical delivery

The use of UAVs to deliver automatic external defibrillators (AEDs) to cardiac arrest victims is a growing area of study, particularly in the USA, Canada, and Sweden [17]. Cardiac events are time-sensitive emergencies in which traditional response times are often insufficient and impacted by system stressors such as staffing shortages [17]. In the case of AED delivery, every minute of delay leads to a 10% increase in mortality [75]. Simulated studies evaluating UAV delivery of AEDs to cardiac arrest patients show a response time advantage compared to emergency responders, with time savings ranging from 1.5 to 15.5 min depending largely on whether UAVs are launched from the emergency response base or from optimized remote bases [17]. In Sweden, UAVs were dispatched to out-of-hospital cardiac arrests and AEDs were successfully delivered in 11 out of 12 eligible alerts, with UAVs arriving before first responders 64% of the time [76]. More limited research has also begun exploring UAV delivery of other emergency products such as intranasal naloxone for opiate overdose, self-injectable EpiPens for anaphylactic shock, or emergency blood products [17].

### Search and rescue

UAVs have been integrated into search and rescue operations in urban, wilderness, combat, and maritime contexts [7, 77]. Preliminary work has explored algorithms to pick out human bodies in standard visual data and in thermal data and to identify other signs of life such as signals from wireless devices or combined approaches [77]. Use of multiple coordinated UAVs also has the potential to improve the efficiency and accuracy of UAV search and rescue efforts [78].

### Incorporation into healthcare management

UAVs can also be incorporated into more complex healthcare management systems. UAVs have been used to collect samples for COVID-19 testing in Ghana through a government contract with Zipline, which was supplemented by philanthropic contributions. Zipline created an efficient transportation system to improve diagnostic access within the constraints of limited laboratory infrastructure [79]. In rural Madagascar, a UAV-based tuberculosis treatment surveillance system was established in 2017. Biannual community health worker surveys identified suspected tuberculosis cases and used UAVs to pick up and transport sputum samples to a diagnostic facility and, in the case of a positive test, deliver 1 month’s worth of medication back to the patient. This approach demonstrated an incremental cost-effectiveness ratio of $177 per disability-adjusted life year (DALY) averted, a measure of disease burden. This cost is well below Madagascar’s per-capita gross domestic product (GDP) threshold, indicating the intervention’s cost effectiveness and health benefits [80]. While still hypothetical, there is interest in expanding the use of UAVs to deliver more complex medical services and collect more extensive samples or health data [81].

One obstacle to expansion in the medical delivery space is the risk of UAV flights in denser urban areas, where there is concern about collision, cybersecurity, and interference with other flight communication networks. At the moment, this means that UAV deliveries may be more feasibly implemented in rural or remote locations, although risks are expected to diminish as technology improves [18]. Public acceptance of UAV use, especially in light of these safety concerns, remains a barrier to widespread adoption as well. UAV deployment in the context of search and rescue purposes, however, has been found to be more widely accepted in the public eye [75]. Robust data collection to assess financial viability, safety, and the impact of successful delivery are needed to more meaningfully integrate UAV technology into the broader medical system.

Across planetary health domains, there are shared opportunities for technical advancements to improve the reliability of UAV deliveries. Areas of focus include improving connectivity consistency, object detection, and resilience to weather conditions as well as reducing inaccuracies in GPS coordinates. Payload limitations are also an obstacle to delivery when blood products or other medical supplies are in high demand [82]. Careful consideration is needed to determine which resources to transport aerially as opposed to using traditional transport methods, balancing the tradeoffs between delivery weight, supply urgency, and the potential quantity that can be delivered. Ultimately, UAVs have the potential to play a lifesaving role in medical delivery, serving a particular niche when ground transport is disrupted, inefficient, or resource-intensive.

## Conclusions

**Fig. 2:**
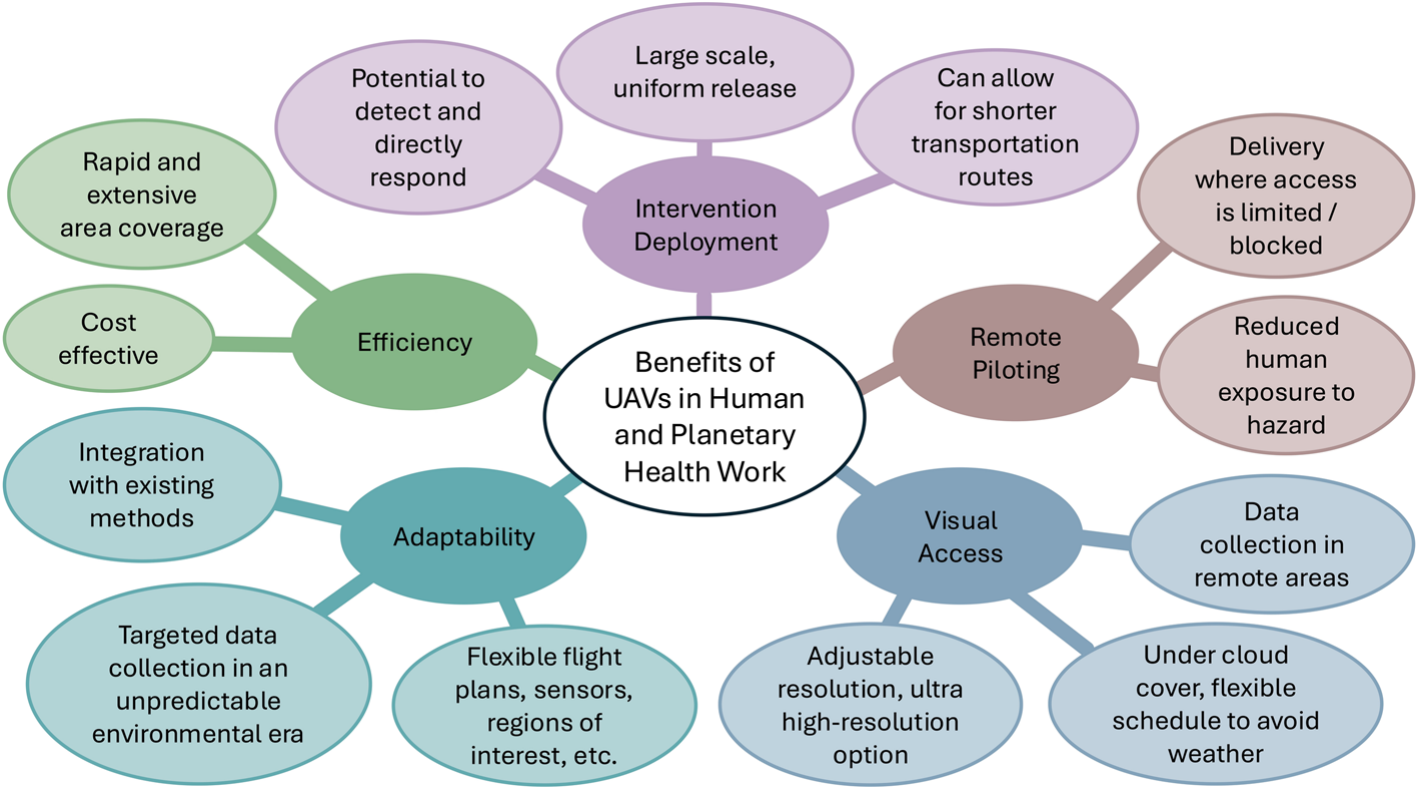
Summary of the key benefits of unmanned aerial vehicle (UAV) use in human and planetary health work. The figure illustrates advantages including efficiency, adaptability to various conditions, broad observational capabilities, and potential for intervention deployment

UAVs are a transformative technology with promising cross-disciplinary applications that can help mitigate the human health impacts of climate change and other environmental disruptions. They provide multiple benefits for human and planetary health work, including rapid response capabilities and cost-effectiveness. Across planetary health applications, we identify five areas in which UAVs offer an advantage: intervention deployment, remote piloting, visual access, adaptability, and efficiency (Fig. 2). These strengths are particularly beneficial in environmental monitoring and health settings in which access or resource constraints limit conventional approaches.

**Fig. 3:**
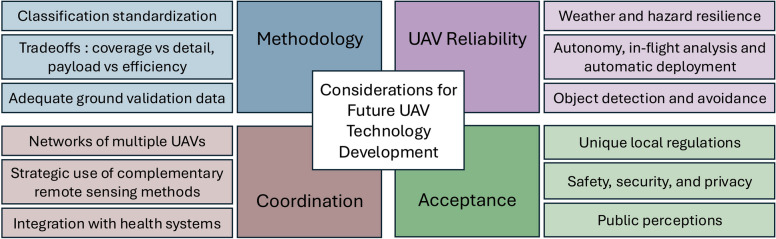
Considerations for future unmanned aerial vehicle (UAV) technology development. The figure outlines critical dimensions including methodology standardization, UAV reliability, coordination networks, and social acceptance factors that should guide future implementation

At the same time, the successful application of UAVs in planetary health requires overcoming challenges that cut across multiple domains. These challenges include the need for standardized procedures, more reliable technology, coordination with other UAVs and sensing networks, and a focus on social acceptance and governance (Fig. 3). Progress in these areas will be critical for scalability and broader adoption of UAV-based approaches in planetary health research and practice. This review is limited by a reliance on published, English-language studies and did not employ a systematic search methodology. Given the field’s rapid pace of technological advancement, particularly with the advent of artificial intelligence (AI), this review provides a snapshot of an evolving field as a foundation for future planetary health work.

UAVs are unique in that they allow us to view our interests, problems, and questions within a broader context but with dynamic and localized detail. They also serve as direct intervention tools, particularly when traditional infrastructure is unreliable. This capability is particularly valuable as we build sustainable systems tailored to our surrounding environments, instead of seeking stability through bigger investments in existing rigid infrastructure. UAVs are also relatively inexpensive and can be operated by community members, opening up potential opportunities for citizen science and widespread use for various monitoring and intervention activities. The broad but flexible perspective facilitated by UAVs is crucial as we tackle global problems that require us to profoundly change our thinking, to understand and value more deeply our role in the health of local and planetary ecosystems, and to create tailored solutions that push us toward sustainability and wellbeing in the future.

## Data Availability

No datasets were generated or analysed during the current study.
